# lncRNA KCNQ1OT1 may function as a competitive endogenous RNA in atrial fibrillation by sponging miR-223-3p

**DOI:** 10.3892/mmr.2021.12510

**Published:** 2021-10-25

**Authors:** Weiran Dai, Xiaoying Chao, Zhiyuan Jiang, Guoqiang Zhong

**Affiliations:** 1 Department of Cardiology, The Second Affiliated Hospital of Chongqing Medical University, Chongqing 400010, P.R. China; 2 Department of Cardiology, The First Affiliated Hospital of Guangxi Medical University, Nanning, Guangxi 530021, P.R. China; 3 Department of Hypertension, The First Affiliated Hospital of Guangxi Medical University, Nanning, Guangxi 530021, P.R. China

**Keywords:** atrial fibrillation, long non-coding RNA KCNQ1 opposite strand/antisense transcript 1, microRNA-223-3p, competing endogenous RNA

## Abstract

Atrial fibrillation (AF) is one of the most common forms of cardiac arrhythmia. Novel evidence has indicated that a competing endogenous RNA (ceRNA) mechanism may occur in AF. The present study aimed to identify differentially expressed microRNAs (miRNAs/miRs) in AF and predict their targeting long non-coding RNAs (lncRNAs) to identify a potential ceRNA network involved in AF using bioinformatics analysis. The GSE68475 microarray dataset was downloaded from the Gene Expression Omnibus database and differentially expressed miRNAs in AF were obtained. In addition, right atrial appendage (RAA) tissues from patients with AF were collected to determine the expression levels of the miRNAs identified following bioinformatics analysis using reverse transcription-quantitative PCR (n=8 per group). Subsequently, Gene Ontology (GO) functional term and Kyoto Encyclopedia of Genes and Genomes (KEGG) signaling pathway enrichment analyses of the target genes of differentially expressed miRNAs of interest were performed. The potential upstream lncRNAs targeting the identified miRNAs were predicted using bioinformatics analysis. A dual luciferase reporter assay was used to verify the existence of a targeted relationship between the differentially expressed miRNA and lncRNA of interest. The results identified 43 differentially expressed miRNAs, including 23 upregulated miRNAs. The trends in the expression levels of miR-223-3p were inconsistent between the microarray data and those recorded in the RAA tissues from patients with persistent AF. Therefore, miR-223-3p was selected as the miRNA of interest for further investigations. The target gene of miR-233-3p was found to be enriched in 57 GO terms and 21 KEGG signaling pathways. According to the bioinformatics prediction, 69 lncRNAs targeting miR-223-3p were identified, including the lncRNA growth arrest-specific transcript 5, lncRNA KCNQ1 opposite strand/antisense transcript 1 (KCNQ1OT1) and lncRNA MYC-induced long non-coding RNA. The results from dual luciferase assay confirmed that miR-223-3p was a direct target of KCNQ1OT1. A ceRNA regulatory relationship may exist between KCNQ1OT1 and miR-223-3p in AF, providing therefore a novel potential research target for further studies.

## Introduction

Atrial fibrillation (AF) is one of the most common forms of cardiac arrhythmia and is accompanied by a high risk of stroke, heart failure and mortality (1). AF is also regarded as a significant contributor towards morbidity and increased healthcare costs (2). The mechanisms underlying AF can be typically classified into three types: Autonomic neural remodeling, electrical remodeling and structural remodeling (3), which can act as triggers or favor the creation of an AF-prone substrate. However, the mechanisms of AF are intricate, which poses challenges for the effective medical intervention of AF.

Recently, multiple molecular factors have been reported to be involved in the pathophysiological progression of AF, including fibrosis, abnormal Ca^2+^ handling and inflammation (4–6). Previous studies have demonstrated the significant role of microRNAs (miRNAs/miRs) in mediating these molecular factors in AF (7). miRNAs are a type of small non-coding RNA of 19–25 nucleotides in length, and which regulate the expression of target genes at the post-transcriptional level by promoting the degradation of target mRNA or repressing target mRNA translation (8). Zhao *et al* (9) reported that miR-29a-3p serves roles in the development of AF by downregulating the L-type Ca^2+^ current (9). Furthermore, Cañón *et al* (10) demonstrated that the aberrant expression of miR-208b could reduce the expression and function of L-type Ca^2+^ channel subunits, as well as the sarcoplasmic reticulum Ca^2+^ pump, ATPase sarcoplasmic/endoplasmic reticulum Ca^2+^ transporting 2, in myocytes isolated from patients with chronic AF, suggesting that miR-208b may be an important mediator in impaired Ca^2+^ handling during atrial remodeling. Our previous study demonstrated that miR-27b-3p could regulate the Wnt/β-catenin signaling pathway and attenuate atrial fibrosis in rats with AF by targeting Wnt3a (11). miRNAs have therefore been considered as promising targets for AF intervention.

Accumulating evidence has indicated that long non-coding RNAs (lncRNAs), a class of non-coding RNAs of >200 nucleotides in length, may also serve an important role in cardiac diseases by acting as competing endogenous RNAs (ceRNAs) (12). Salmena *et al* (13) first proposed the ceRNA theory in 2011, and hypothesized that mRNAs and lncRNAs could communicate with each other by binding to shared miRNAs using miRNA response elements (14). For example, the lncRNA-long intergenic non-protein coding RNA 472 was discovered to promote AF by downregulating the expression of ryanodine receptor 2 via miR-24 (15). However, the underlying regulatory ceRNA mechanism in AF remains to be fully elucidated.

The present study firstly aimed to identify significantly differentially expressed miRNAs in AF by screening microarray data from the Gene Expression Omnibus (GEO) database. Then, right atrial appendage (RAA) tissues from patients with AF were collected to verify the expression of the identified miRNAs. Combined with the validation results, Gene Ontology (GO) functional term and Kyoto Encyclopedia of Genes and Genomes (KEGG) signaling pathway enrichment analyses were performed for the target genes of the differentially expressed miRNAs of interest to fully understand the role of these miRNAs. The potential upstream lncRNAs targeting the identified miRNAs were predicted using Bioinformatics method. Finally, a dual luciferase assay was used to verify whether a targeted relationship existed between significantly differentially expressed miRNAs and lncRNAs of interest. These findings may provide a solid theoretical basis for future research to help identifying potential targets for the treatment of AF.

## Materials and methods

### Microarray data collection

The present study used the GEO database (https://www.ncbi.nlm.nih.gov/geo), which is a public gene expression database from the National Center for Biotechnology Information. For microarray retrieval, ‘atrial fibrillation’ was used as the key word to search for microarrays that had studied the expression levels of miRNAs in AF. The inclusion criteria were as follows: i) Human atrial appendage tissue as the research sample type; ii) the Sinus rhythm (SR) group was set as the control group and the persistent AF group was set as the experimental group, and each group contained >3 research samples; and iii) miRNA expression in atrial appendage tissues was the main research focus. The exclusion criteria were as follows: i) Incomplete microarray matrix data; ii) the source of the research sample was unclear; iii) the main research focus of the microarray matrix was not miRNAs; iv) the microarray annotation file was missing or unknown; and v) the microarray data were unable to meet the subsequent data analysis standards after standardized processing. After retrieval, the platform files and microarray data that met the criteria were downloaded for subsequent analysis.

### Data preprocessing

To reduce the errors in data analysis, data preprocessing of the downloaded microarray data was performed. Firstly, the corresponding microarray platform files were used to annotate the matrix data of the microarray and convert the miRNA IDs. After annotation, the annotated matrix data were supplemented using the impute toolkit of R software (version 3.5.1; RStudio, Inc.). All matrix data that did not match the miRNAs were deleted. For the matrix data with the same miRNA ID, the average value was obtained after merging. Finally, Bioconductor's limma toolkit (version 3.10.3; http://www.bioconductor.org/packages/2.9/bioc/html/limma.html) in R software was used for background correction, provision of missing values and data standardization.

### Identification of differentially expressed miRNAs

After data preprocessing, the limma toolkit in R software was used to identify the significantly differentially expressed miRNAs between AF and SR samples. The screening threshold of significantly differentially expressed miRNAs was set as |log_2_ fold change (FC)| ≥0.5 and P<0.05. The heatmap and volcano map of significantly differentially expressed miRNAs were drawn using R software. The top four most significantly upregulated and downregulated miRNAs, according to the |log_2_FC| values, were selected for verification using reverse transcription-quantitative PCR (RT-qPCR).

### Patient studies

We continuously included patients with SR and persistent AF who were hospitalized at the First Affiliated Hospital of Guangxi Medical University (Nanning, China) and were undergoing open-heart surgery for coronary bypass grafting or valve replacement. The patient's electrocardiogram was independently judged by two cardiovascular physicians and combined with medical history. The diagnostic criteria of AF was in line with the diagnostic criteria of AF recommended in the 2016 European Society of Cardiology AF management guidelines (16). Patients were excluded if they had the additional following conditions: left ventricular ejection fraction <50%, infectious diseases, active myocarditis, endocarditis, active rheumatism, pulmonary disease, hyperthyroidism, hematological diseases, tumors and autoimmune diseases. Since September 2018, RAA tissues were collected at the beginning of surgical intervention under extracorporeal circulation and rapidly cryopreserved in liquid nitrogen, then stored at −80°C until the RAA tissue of the last patient was collected in June 2019. All samples would then be used for RT-qPCR analysis. The experimental protocol was approved by the Ethics Committee of The First Affiliated Hospital of Guangxi Medical University (Nanning, China). Each patient provided written informed consent prior to surgery for RAA tissue collection.

### RT-qPCR

Total RNA was extracted from the RAA tissues from patients with AF or SR (n=8 per group) using TRIzol^®^ reagent (cat. no. 9108; Takara Bio, Inc.). A NanoDrop ND 1000 spectrophotometer was used to assess the absorbance of the extracted total RNA at wavelengths of 260 and 280 nm. The 260/280 ratio was calculated to confirm the quality of the RNA, and only samples with a 260/280 ratio between 1.8 and 2.1 were used. Total RNA was reverse transcribed into cDNA using a tailing reaction kit (cat. no. B532451; Sangon Biotech Co., Ltd.) for miRNA or a reverse transcription kit (cat. no. RR047A; Takara Bio, Inc.) for mRNA according to the manufacturers' instructions. When the universal reverse primers provided by the tailing reaction kit could not complete the cDNA synthesis of miRNA experiment, we replaced the universal reverse primers or redesigned the reverse primers to complete this part of the study. qPCR was subsequently performed on a StepOne Real-Time PCR system (Thermo Fisher Scientific, Inc.). The PCR procedure was as follows: One cycle at 95°C for 30 sec to complete the initial denaturation, followed by 40 cycles of 95°C for 5 sec and 60°C for 30 sec for denaturation, annealing and extension. Melting curve analysis was performed at ~65–95°C. Expression levels were quantified using the 2^−∆∆Cq^ method (17) and normalized to GAPDH (for mRNA) or U6 (for miRNA). The sequences of the primers used are listed in Table I.

### Bioinformatics analysis

By combining the data from the microarray analysis and patient tissues, the differentially expressed miRNAs of interest were selected for bioinformatics analysis to further explore their functions. GO functional term and KEGG signaling pathway enrichment analyses were conducted for the target genes of the differentially expressed miRNAs of interest (18,19). The GO functional term enrichment analysis consisted of biological process (BP), cellular component (CC) and molecular function (MF) aspects. P<0.05 and a count value ≥2 were used as the significant cutoff values. The results from the GO functional term and KEGG signaling pathway enrichment analyses were sorted using the Revigo toolkit in R software and visualized in the form of bubble charts.

### Prediction of lncRNAs targeting the differentially expressed miRNAs of interest

The miRbase (http://www.mirbase.org) and StarBase (http://starbase.sysu.edu.cn/index.php) databases were used to predict the upstream lncRNAs targeting the differentially expressed miRNAs of interest. The identified lncRNAs were inputted into Cytoscape software (version 3.7.2) to construct the ceRNA network.

### Dual luciferase reporter assay

The recombinant luciferase construct, pcDNA3.1-KCNQ1OT1 3′-untranslated region (UTR)-wild-type (WT; Hanbio Biotechnology Co., Ltd.) harboring the WT binding site for miR-223-3p and another recombinant construct, pcDNA3.1-KCNQ1OT1 3′-UTR-mutant (MUT), containing the mutated binding site for miR-223-3p, were constructed. Then, 293T cells (The Cell Bank of Type Culture Collection of The Chinese Academy of Sciences) were co-transfected with the pcDNA3.1-KCNQ1OT1 3′-UTR-WT (KCNQ1OT1 WT) or pcDNA3.1-KCNQ1OT1 3′-UTR-MUT (KCNQ1OT1 MUT) recombinant plasmid and miR-223-3p mimic (sense 5′-UGUCAGUUUGUCAAAUACCCC-3′, antisense 5′-GGGGUAUUUGACAAACUGACA-3′) or miR-223-3p mimic-negative control (sense 5′-UUUGUACUACACAAAAGUACUG-3′, antisense 5′-CAGUACUUUUGUGUAGUACAAA-3′) by Liposomal Transfection Reagent (Hanbio Biotechnology Co., Ltd.). The miR-223-3p mimic and miR-223-3p mimic-negative control were constructed by GenePharma Co., Ltd. After 48 h of transfection, the Dual-Luciferase Reporter Assay system (cat. no. E1910; Promega Corporation) was used to measure the relative luciferase activity in each group and the data normalization was performed by comparing with the *Renilla* luciferase activity of KCNQ1OT1 WT + miR-223-3p mimic group.

### Statistical analysis

Statistical analysis was performed using SPSS version 23 software (IBM Corp.). The categorical data are presented as the frequency and the continuous data are presented as the means ± standard deviation. A χ^2^ test was used to determine the statistical differences between categorical data. For continuous and normally distributed data, statistical differences between two groups were compared using a unpaired two-tailed Student's t-test. P<0.05 was considered to indicate a statistically significant difference.

## Results

### Microarray data

After screening, the miRNA microarray dataset GSE68475, which contained 10 patients with AF and 11 patients with SR, was selected for further analysis. The GSE68475 dataset, which was uploaded by Morishima *et al* (20), aimed to detect the miRNAs expression in human atrial appendages, and reported that miR-30d was essential for the electrical remodeling of AF. After preprocessing the miRNA microarray data according to the degree of samples dispersion, two samples from both the AF and SR groups were removed. Finally, eight samples from the AF group and nine samples from the SR group were obtained.

### Identification of differentially expressed miRNAs in AF

**Figure 1.**
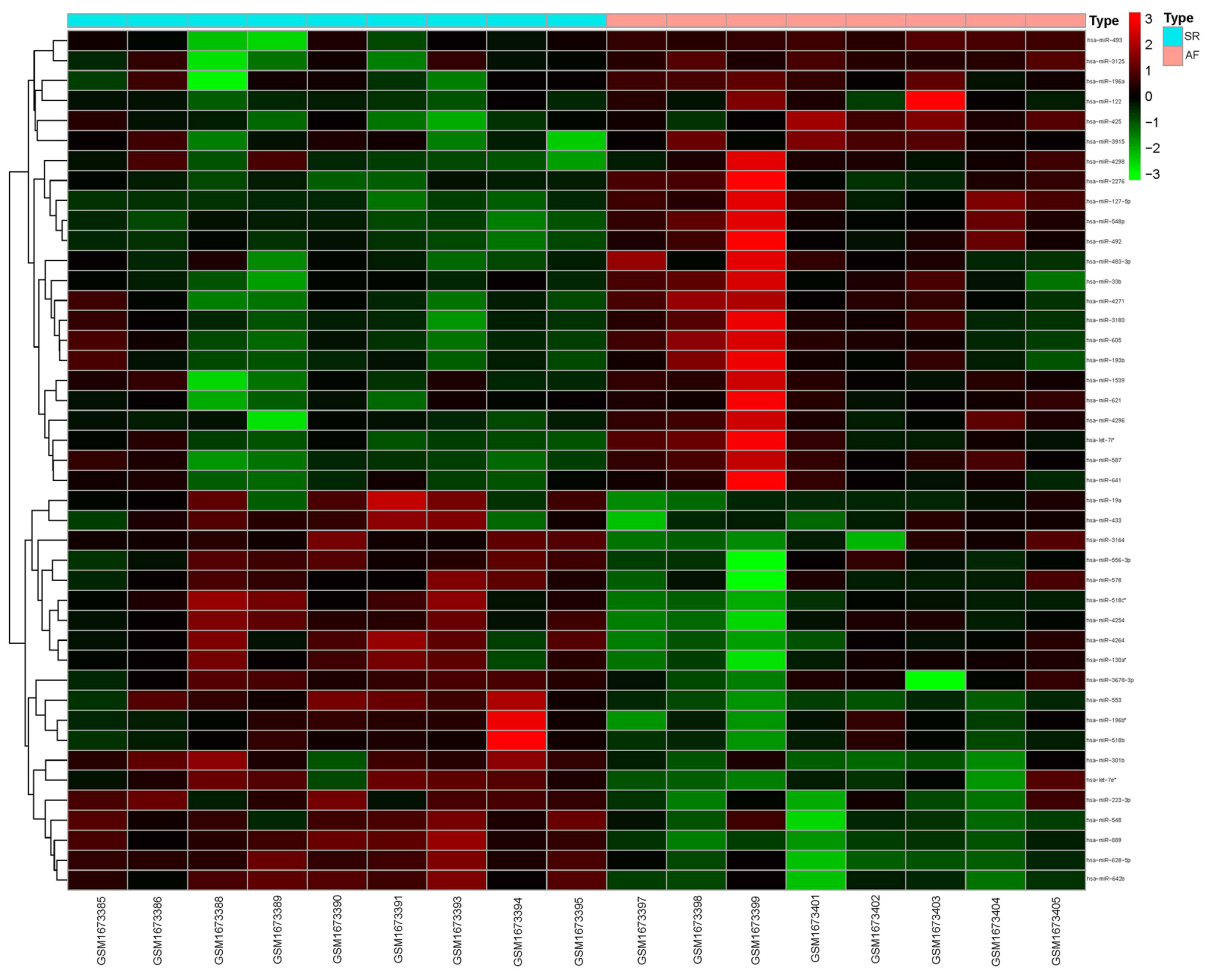
Heatmap of significantly differentially expressed microRNAs in AF. SR, sinus rhythm; AF, atrial fibrillation.

**Figure 2.**
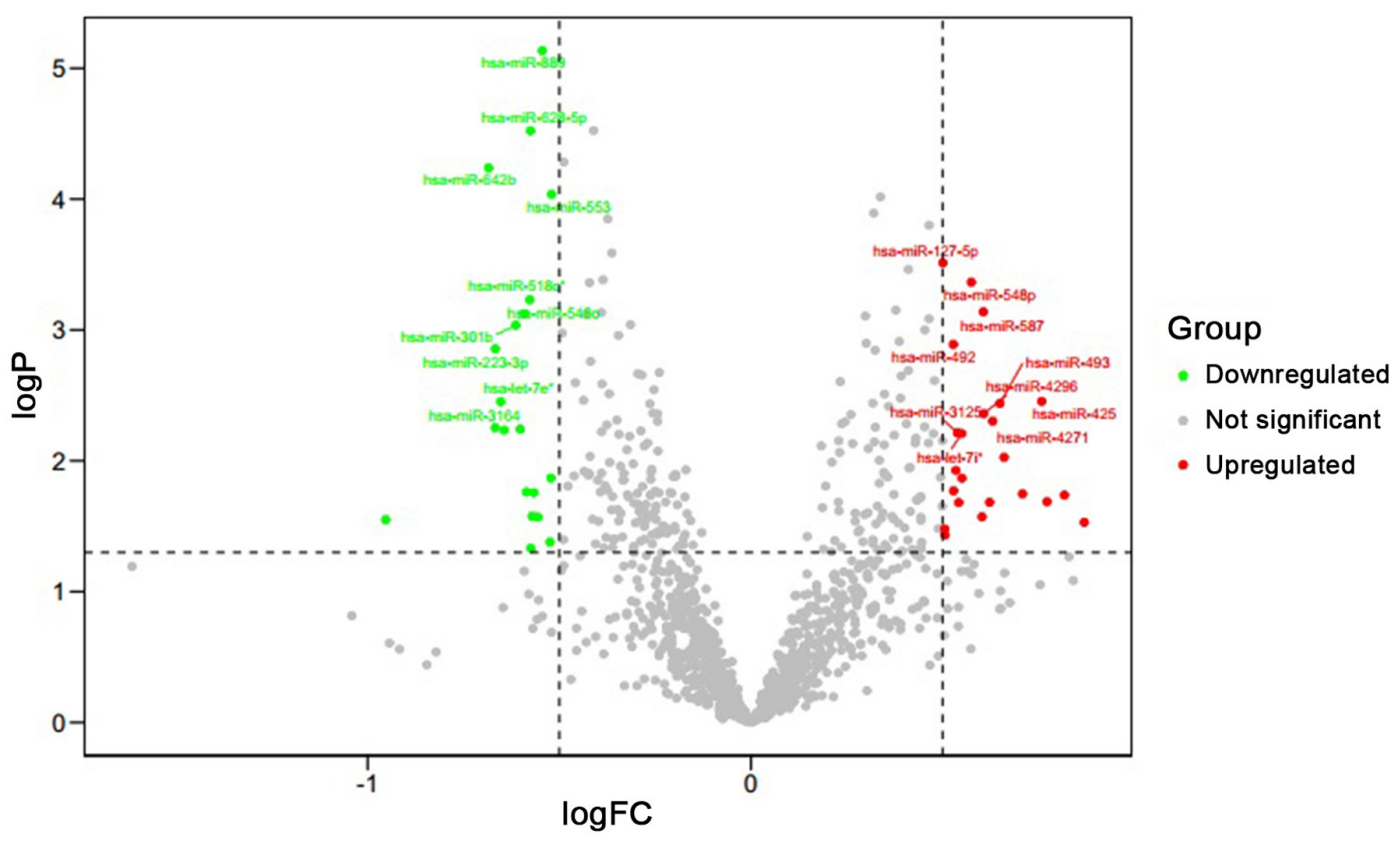
Volcano map of significantly differentially expressed miRNAs in AF. Green points show the downregulated miRNAs and red points show the upregulated miRNAs in AF samples. AF, atrial fibrillation; FC, fold change; miRNAs, microRNAs.

miRNAs were screened for according to the threshold cutoff values used in the present study. The results identified 43 differentially expressed miRNAs in the AF and SR groups, including 23 upregulated miRNAs, such as miR-33b, miR-483-3p, miR-122 and miR-425 (Table II), and 20 downregulated miRNAs, such as miR-196b, miR-642b, miR-3164 and miR-223-3p (Table III). The heatmap and volcano map displaying the differentially expressed miRNAs were drawn using R software and are presented in Figs. 1 and 2.

### Identification of differentially expressed miRNAs in RAA tissues from patients with AF

To verify the expression of the aforementioned differentially expressed miRNAs identified by screening the microarray data, RT-qPCR was used to determine the expression levels of the identified miRNAs in the RAA tissues from patients with AF or SR. In total, 16 patients (age range, 42–69 years) were used, including eight patients with persistent AF. The clinicopathological characteristics of the included patients are listed in Table IV, and no significant differences were observed in the sex, age, medical history or therapeutic regimen between the two groups.

**Figure 3.**
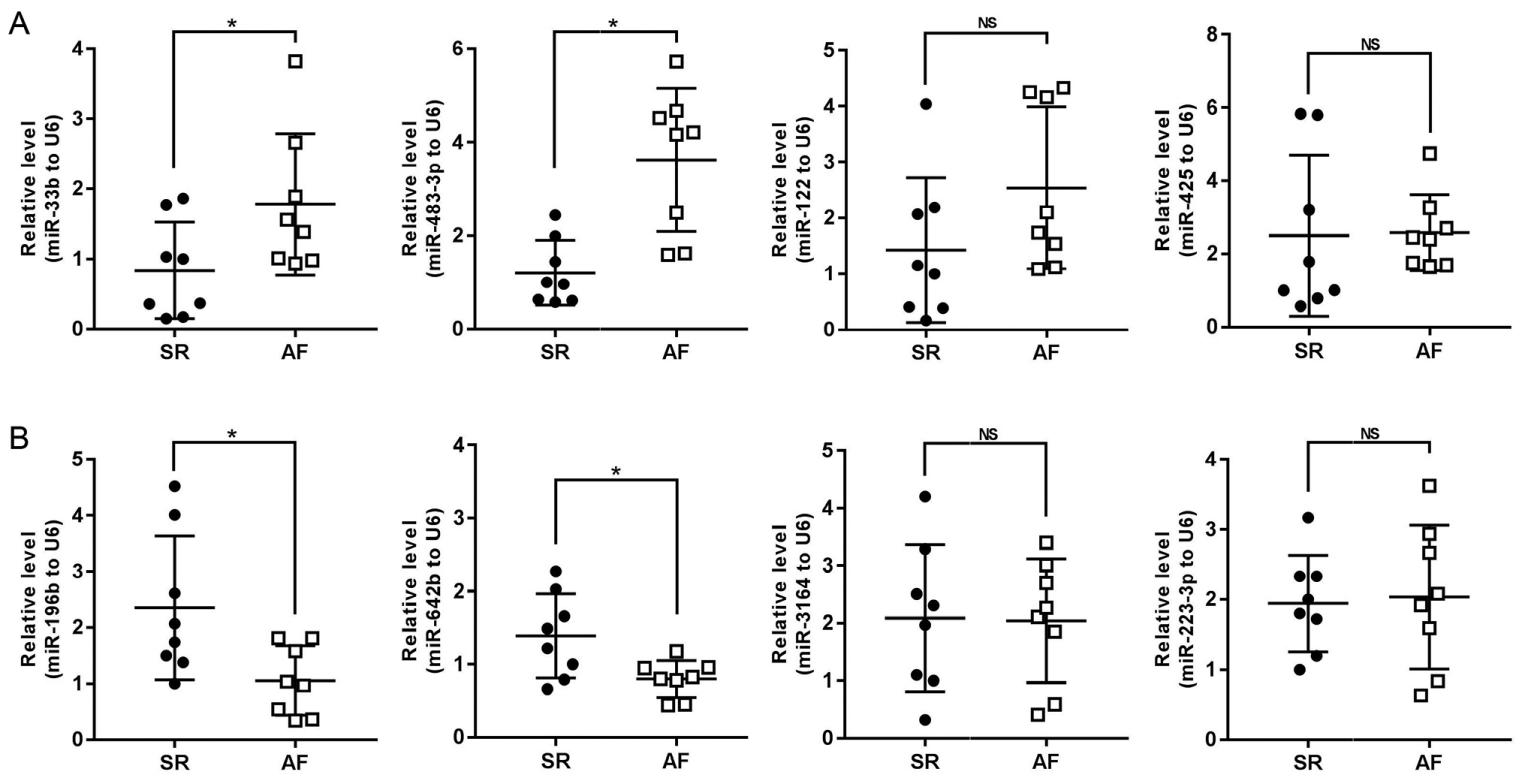
Expression of differentially expressed miRNAs selected from microarray were verified in right atrial appendage of patients with SR or chronic AF by RT-qPCR. (A) First four upregulated miRNAs were verified by RT-qPCR (n=8 per group). (B) First four downregulated miRNAs were verified by RT-qPCR (n=8 per group). *P<0.05 vs. SR group. SR, sinus rhythm; AF, atrial fibrillation; NS, non-significant; RT-qPCR, reverse transcription quantitative PCR; miRNAs, microRNAs.

Compared with patients with SR, the expression levels of miR-33b and miR-483-3p were found to be upregulated in the AF group, while the expression levels of miR-196b and miR-642b were downregulated. These findings were consistent with the microarray data. However, no significant differences were identified in the expression levels of miR-122, miR-425, miR-3164 and miR-223-3p between the two groups (Fig. 3). Our previous study reported that miR-223-3p could mediate the generation of angiotensin (Ang)II-induced reactive oxygen species and thereby regulate the levels of oxidative stress (Weiran Dai; unpublished data). Oxidative stress is one of the important mechanisms required for the initiation and maintenance of AF. Zhang *et al* (21) reported that miR-223-3p regulates the activation of paired box 6, resulting in apoptosis, a mechanism that plays a significant role in the progression of the prothrombotic state in AF. Novel evidence has suggested that the inhibition of miR-223-3p expression could upregulate the expression of FOXO3 and activate the autophagy pathway, thereby significantly inhibiting myocardial fibrosis and improving myocardial remodeling in AF (22). miR-223-3p may therefore have multiple regulatory mechanisms in AF and understanding the biological function and specific regulatory mechanisms of miR-223-3p in AF may be of great significance. For these reasons, miR-223-3p was selected for further analysis in the present study.

### Functional enrichment analyses for target genes of miR-223-3p

**Figure 4.**
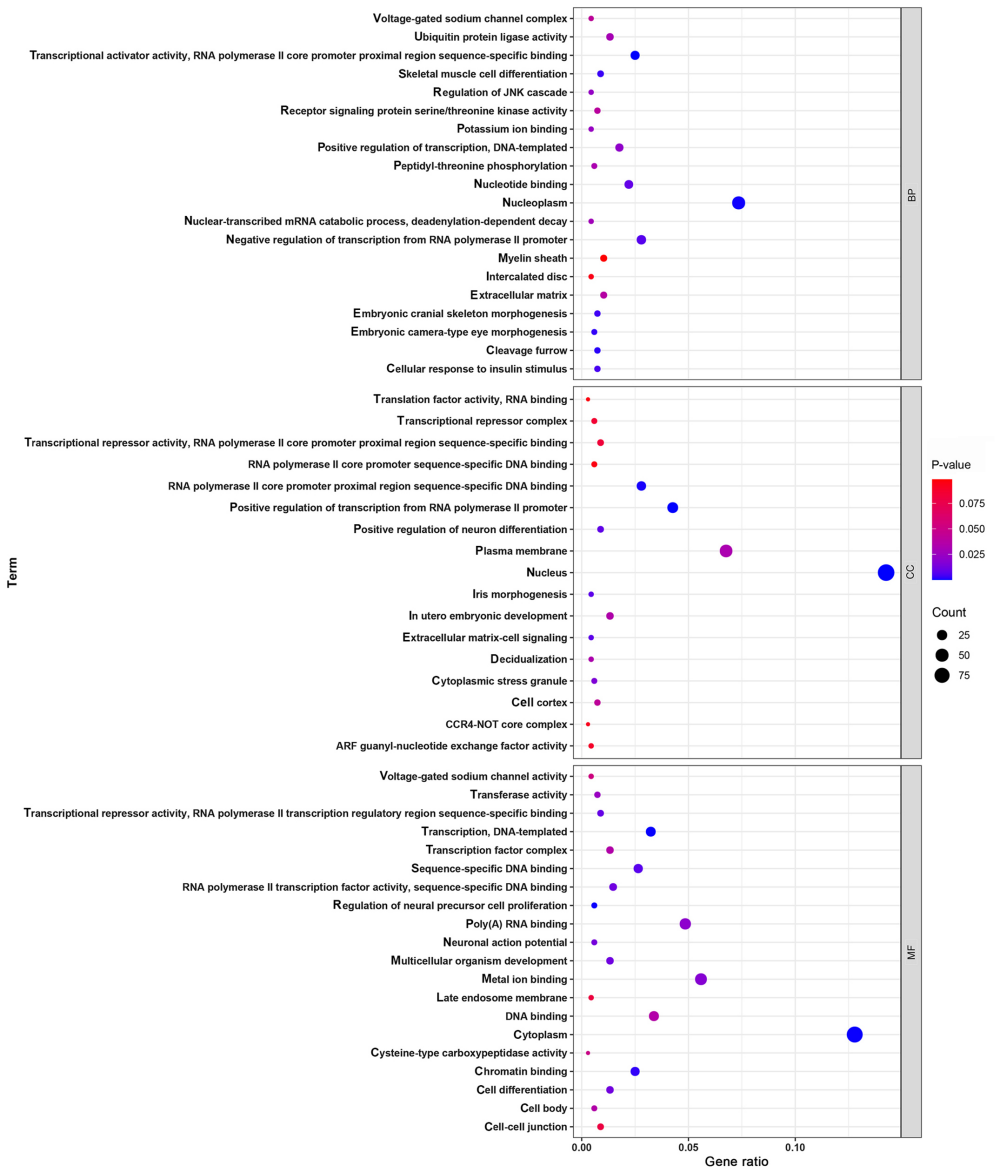
Gene Ontology function enrichment analysis of microRNA-223-3p target genes. BP, biological process; CC, cellular component; MF, molecular function.

**Figure 5.**
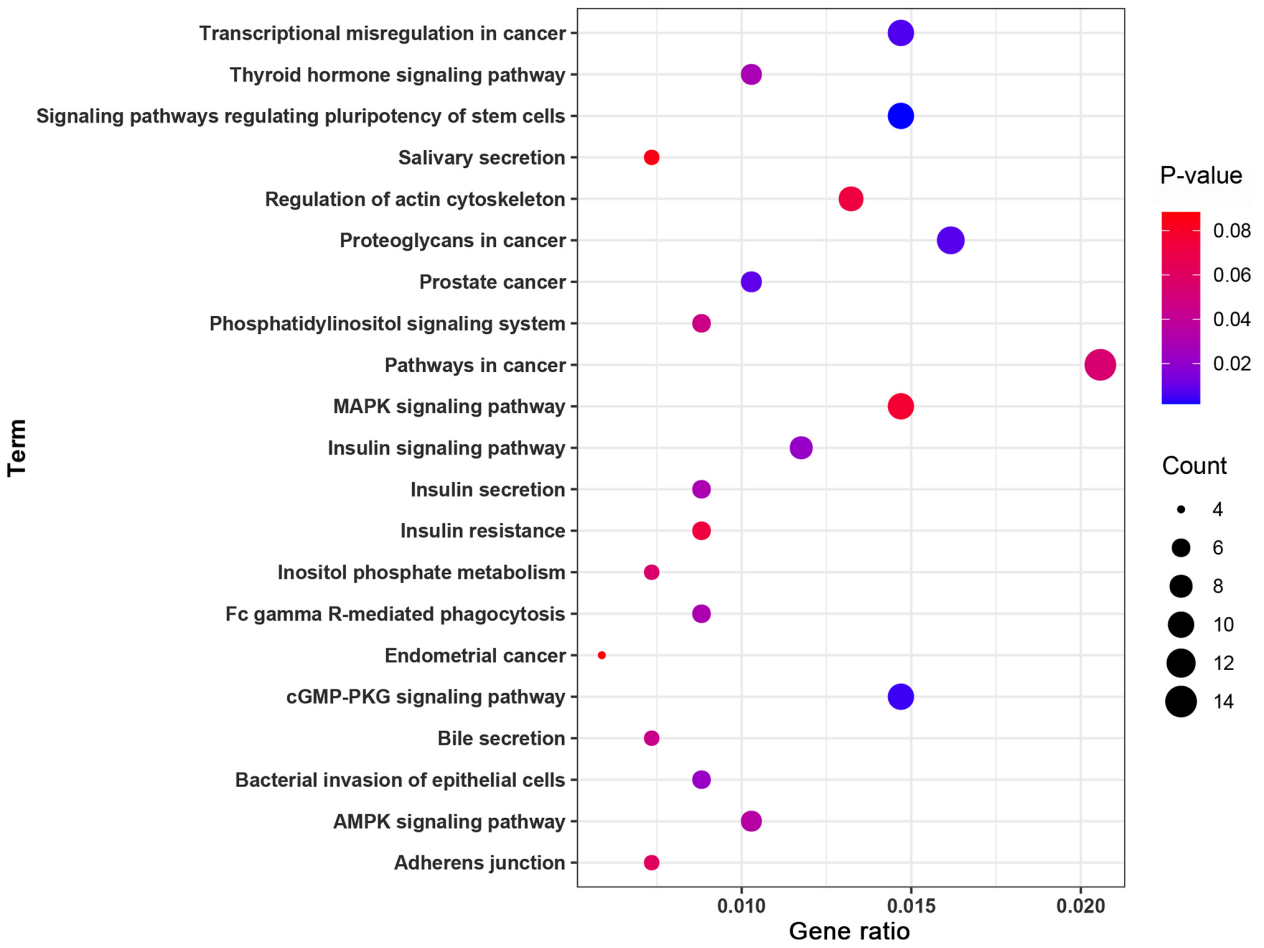
Kyoto Encyclopedia of Genes and Genomes pathway enrichment analysis of the target genes of microRNA-223-3p.

According to the predictions of the bioinformatics database, 415 target genes were identified as having a potential regulatory relationship with miR-223-3p. To analyze the potential biological functions of target genes of miR-223-3p, GO functional terms and KEGG signaling pathways enriched by the target genes of miR-223-3p were identified using the Database for Annotation, Visualization and Integrated Discovery (version 6.8; http://david.ncifcrf.gov/). A total of 57 GO functional terms and 21 KEGG signaling pathways were obtained. For GO enrichment analysis, target genes of miR-223-3p were enriched in 20 BP, 17 CC and 20 MF, including ‘nuclear function’, ‘transcription factor activity regulation’, ‘RNA polymerase II promoter’, ‘DNA template translation’ and ‘cytoplasmic composition’ (Fig. 4). The results from the KEGG signaling pathway enrichment analysis revealed that target genes of miR-223-3p were enriched in 21 KEGG signaling pathways, including ‘AMPK signaling pathway’, ‘stem cell pluripotency regulation’, ‘cGMP PKG signaling pathway’ and ‘insulin signaling pathway’ (Fig. 5).

### Identification of lncRNAs targeting miR-223-3p in the RAA tissues from patients with AF

**Figure 6.**
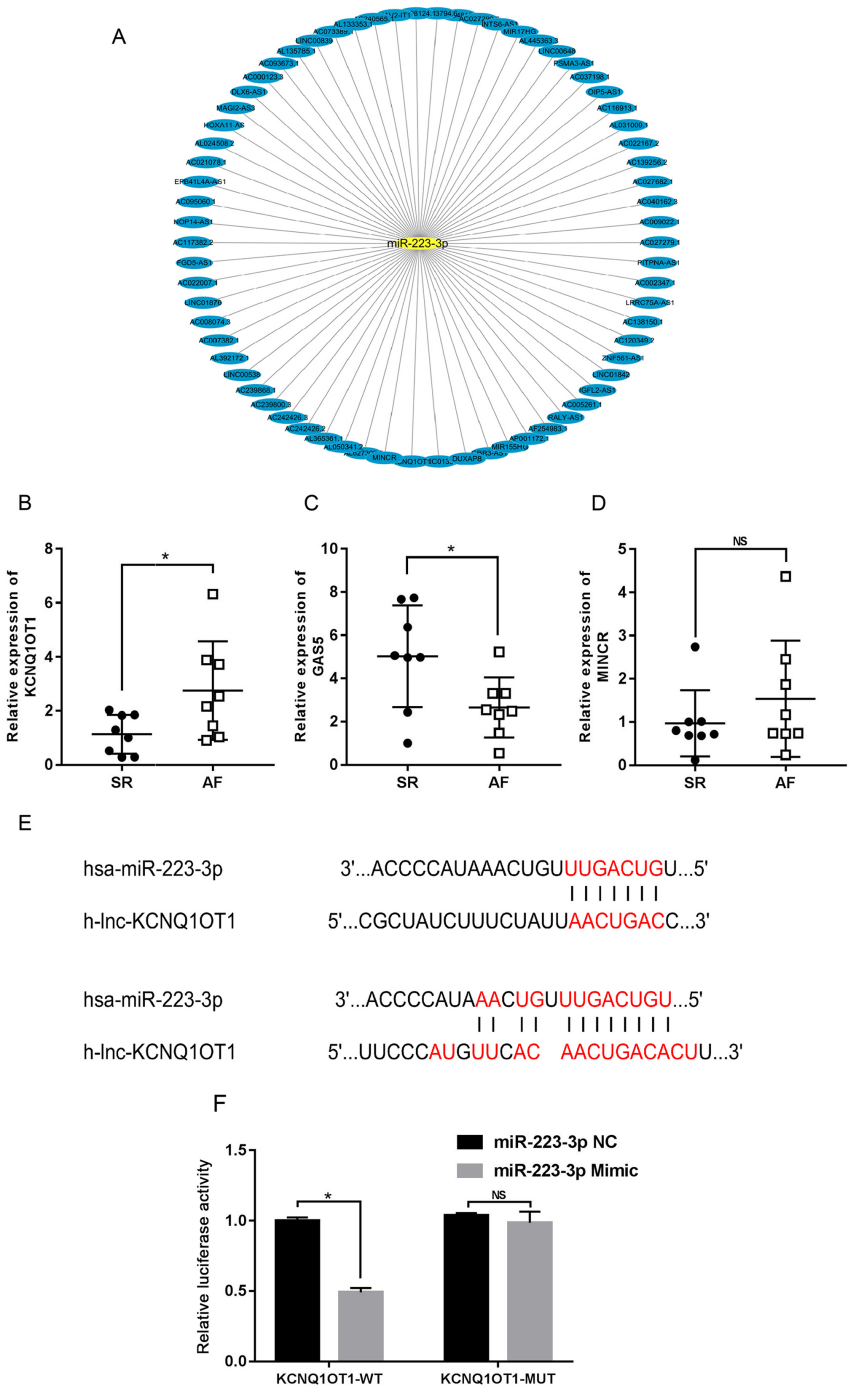
miR-223-3p is one direct target of KCNQ1OT1. (A) Bioinformatics database predicted the lncRNAs targeting miR-223-3p. (B-D) Expression of KCNQ1OT1, GAS5 and MINCR in RAA of patients with SR or AF (n=8 per group); *P<0.05 vs. SR group. (E) Binding site of KCNQ1OT1 and miR-223-3p. (F) Dual luciferase assay confirmed that miR-223-3p was one direct target of KCNQ1OT1 (n=3 per group). *P<0.05 vs. KCNQ1OT1 WT + miR-223-3p NC group. SR, sinus rhythm; AF, atrial fibrillation; RAA, right atrial appendage; NS, non-significant; WT, wild type; MUT, mutant; miR, microRNA; NC, negative control; KCNQ1OT1, KCNQ1 opposite strand/antisense transcript 1.

According to the bioinformatics database prediction, 69 lncRNAs targeting miR-223-3p were identified, including lncRNA growth arrest specific transcript 5 (GAS5), lncRNA KCNQ1 opposite strand/antisense transcript 1 (KCNQ1OT1) and lncRNA MYC-induced long non-coding RNA (MINCR; Fig. 6A). As presented in Fig. 6B, the results from RT-qPCR analysis revealed that, compared with SR group, the expression level of KCNQ1OT1 was upregulated in RAA tissues from patients with AF. Conversely, the expression level of GAS5 was lower in patients with AF compared with that in patients with SR (Fig. 6C). However, no significant differences in the expression level of MINCR were identified between the two groups (P>0.05; Fig. 6D). Furthermore, KCNQ1OT1 was identified as an AF-related lncRNA using GeneCards (https://www.genecards.org). KCNQ1OT1 was therefore selected for further analysis in the present study.

### miR-223-3p is a direct target of KCNQ1OT1

To further investigate whether KCNQ1OT1 may directly mediate miR-223-3p, a dual luciferase reporter assay was performed. The results from the bioinformatics analysis suggested that miR-223-3p may be considered as a potential target of KCNQ1OT1, with two conserved putative binding sites identified between miR-223-3p and KCNQ1OT1 (Fig. 6E). The results from the dual luciferase reporter assay demonstrated that, compared with the KCNQ1OT1 WT + miR-223-3p mimic-NC group, the relative luciferase activity of the KCNQ1OT1 WT + miR-223-3p mimic group was significantly decreased (1.0102±0.0234 vs. 0.4903±0.0316), while the decrease in relative luciferase activity was reversed in the KCNQ1OT1 MUT + miR-223-3p mimic and KCNQ1OT1 MUT + miR-223-3p mimic-NC groups (Fig. 6F). Taken together, these results suggested that miR-223-3p may be a direct target of KCNQ1OT1, and that a ceRNA regulatory relationship may exist between KCNQ1OT1 and miR-223-3p in AF. These findings provided a novel potential target for the treatment of AF and might initiate further investigations.

## Discussion

Despite the significant progress that has been made in the treatment of AF, AF continues to negatively impact individuals' lives, and the incidence increases with age. A recent study suggested that lncRNAs function as ceRNAs and could regulate miRNA expression and thereby affect the expression of target genes of miRNAs to contribute to the pathogenesis of AF (23). Therefore, screening for differentially expressed miRNAs using bioinformatics analysis remains an effective method to identify potential targets for future research into AF intervention. To the best of our knowledge, the present study was the first to identify and verify the specific regulatory relationship between KCNQ1OT1 and miR-223-3p using microarray data and may provide a promising target for future AF intervention experiments.

In cardiology, miR-223-3p has been shown to exert multiple regulatory effects. Previous studies have reported that miR-223-3p can regulate cell proliferation, apoptosis and necrosis at the post-transcriptional level via targeted inhibition mechanisms, and can also exert anti-inflammatory effects (24–26). A previous study reported that miR-223-3p could directly inhibit IκB kinase α to regulate the inflammatory response and necrosis induced by myocardial ischemia/reperfusion injury (27). Furthermore, another study suggested that circulating miR-223-3p levels might be used as a circulating marker of unstable plaques in coronary atherosclerotic heart disease (28). To fully understand the biological function of miR-223-3p, the present study performed GO functional term and KEGG signaling pathway enrichment analyses for potential miR-223-3p target genes. The results from the GO functional term enrichment analysis revealed that target genes were mainly enriched in ‘nuclear function’, ‘transcription factor activity regulation’, ‘RNA polymerase II promoter’, ‘DNA template translation’ and ‘cytoplasmic composition’. The results from the KEGG signaling pathway enrichment analysis demonstrated that target genes were mainly enriched in the ‘AMPK signaling pathway’, ‘stem cell pluripotency regulation’, ‘cGMP PKG signaling pathway’ and ‘insulin signaling pathway’.

The AMPK signaling pathway is an important energy metabolism pathway, which plays an essential role in the regulation of cardiomyocyte energy homeostasis. AMPK regulates the metabolism of fatty acids and glycogen to maintain energy balance within the cell. Previous evidence also revealed that AMPK could regulate protein synthesis and cell proliferation via the eukaryotic translation elongation factor 2 and TSC complex subunit 2/mTOR signaling pathways and regulate blood flow via endothelial or neuronal nitric oxide synthase (29). In addition, the AMPK signaling pathway is also considered as a cardiac metabolite regulator. AMPK is a sensitive sensor of cell stress, which can be activated by phosphorylation of the upstream signal at position 172 threonine, and then exerts a protective effect by preventing metabolic stress, cell proliferation and hypertrophic remodeling, and regulating membrane electrophysiology. In addition, a previous study demonstrated that AMPK can also regulates the expression levels of Ca^2+^ channels and ion transporters to mediate intracellular Ca^2+^ signaling processing and transmission (30). In addition, abnormal Ca^2+^ handling may affect the action potential duration, which is an important mechanism of AF induction. Harada *et al* (31) report that downregulation of AMPK inhibits the abnormal Ca^2+^ transmission mediated by the integrity of atrial L-type Ca^2+^ channels, suggesting that the AMPK signaling pathway might play an important role in maintaining cardiac ion homeostasis and arrhythmias, including AF. Amongst the susceptibility models of AF, collagen-induced arthritis (CIA) is associated with the increased expression of inflammatory cytokines (interleukin-6 and tumor necrosis factor-α), and it has been shown to lead to atrial remodeling and AF. Zhang *et al* (32) used a CIA rat model and demonstrated that CIA could induce atrial energy metabolism disorder by inhibiting the AMPK/PPARG coactivator 1 α signaling pathway, while resveratrol could effectively reverse this inhibitory effect and reduce the susceptibility of AF in CIA model rats, suggesting that the increased risk of AF may be associated with the downregulation of the AMPK signaling pathway (33,34). Another study demonstrated that fisetin can promote AMPK phosphorylation in the atrium, reducing AF susceptibility following myocardial infarction (35). The AMPK signaling pathway may therefore represent a potential research direction for future studies into AF intervention.

lncRNAs are important upstream regulators of the miRNA-associated ceRNA network by sponging miRNAs with related binding sites. In the present study, bioinformatics analysis predicted that 69 lncRNAs could target miR-223-3p, including GAS5, KCNQ1OT1 and MINCR. The expression of these lncRNAs was subsequently verified in RAA tissues from patients with AF, and the results demonstrated that compared with patients with SR, KCNQ1OT1 was upregulated and GAS5 was downregulated.

KCNQ1OT1 is located in the 15.5 region of the KCNQ1 locus on chromosome 11 (36). KCNQ1OT1 has been reported to be associated with numerous types of disease, including cardiovascular diseases (37). Li *et al* (38) demonstrated that KCNQ1OT1 could prevent myocardial ischemia/reperfusion injury following acute myocardial infarction by regulating adiponectin receptor 1. Furthermore, KCNQ1OT1 was identified as an AF-related lncRNA using the GeneCard database. Consistent with the results from the present study, Shen *et al* (39) reported that KCNQ1OT1 expression is significantly upregulated in a time-dependent manner in an angiotensin II (AngII) infusion mouse model. AngII treatment has been demonstrated to effectively increase AF susceptibility. AngII is therefore widely used in scientific research to establish an AF susceptibility model. In addition, KCNQ1OT1 was shown to be an essential lncRNA involved in the regulation of a cardiovascular disease ceRNA network, and it could function as a molecular sponge by binding to miRNAs to regulate the expression of their downstream target genes. Yang *et al* (40) report that KCNQ1OT1 is highly expressed in patients with diabetic cardiomyopathy and that silencing KCNQ1OT1 successfully inhibits cell apoptosis by increasing the expression of miR-214-3p and then downregulating its target gene by acting as a ceRNA *in vivo*. Furthermore, a previous study indicated that KCNQ1OT1 might be involved in the occurrence of AF as a ceRNA by mediating the miR-384b/Ca^2+^ voltage-gated channel subunit α 1C axis (39). In the present study, the results from the dual luciferase reporter assay revealed that miR-223-3p could directly bind to KCNQ1OT1, suggesting that KCNQ1OT1 may regulate the expression of downstream genes targeted by miR-223-3p to exert its biological molecular effects. The findings from the present study may therefore provide a new promising target for AF intervention.

The lncRNA GAS5 plays an important role in a variety of cardiovascular diseases. For example, in hypertension, GAS5 has been considered as a new vascular remodeling regulator (41). In AF, GAS5 can reverse the proliferation of cardiac fibroblasts by inhibiting transforming growth factor β receptor 1, thus decreasing the synthesis of collagen fibers in the extracellular matrix and inhibiting the structural remodeling of the myocardium and the progression of AF (42). In addition, GAS5 has also been reported to serve as a ceRNA in cardiovascular diseases and inhibit the negative regulation of miRNAs on downstream gene expression. Zhou *et al* (43) demonstrated that GAS5, by acting as a ceRNA, can inhibit miR-21 via its molecular sponge action, thus regulating the expression of programmed cell death 4, a direct target of miR-21, and mediating cell apoptosis in myocardial infarction. Increasing evidence has suggested that GAS5 might also act as a ceRNA and inhibit miR-223-3p, thereby regulating the expression of its downstream target genes (44). Similarly, Yao *et al* (45) reported that miR-223-3p could directly bind to the 3′-UTR of nicotinamide phosphoribosyltransferase (NAMPT) and that GAS5 could sponge miR-223-3p and relieve the inhibitory effect of miR-223-3p on NAMPT via regulating PI3K/AKT signaling. As there have been numerous studies demonstrating that GAS5 can directly target miR-223-3p (44,45), the present study did not perform dual luciferase reporter assays to verify whether miR-223-3p was a target of GAS5. However, the underlying mechanism of GAS5 in AF based on the ceRNA theory has not been studied in detail, to the best of our knowledge, and will require further experimental verification.

The results from the present study provided novel promising targets for further experimentation to develop strategies for AF intervention. However, there were some limitations to the present study. Firstly, human samples were used to verify the differentially expressed miRNAs of interest found within the microarray dataset, in addition to the trends in the expression levels of upstream lncRNAs in AF. Unfortunately, the study did not perform *in vitro* transfection experiments to further verify the direct targeted regulatory relationship between the identified miRNAs and lncRNAs. Hence, the lack of functional *in vitro* experiments was a limitation to this study. The author's research group is currently performing *in vivo* and *in vitro* experiments to validate the results from the present study. Secondly, the sample size used in this study was relatively small, which may produce selection offset. Some evidence has suggested that miR-223-3p expression in AF is controversial. For example, Wang *et al* (46) studied the miRNA transcriptome in AF using a tissue microarray and demonstrated that miR-223-3p expression level is upregulated in AF tissues. Conversely, a recent study using exosomes isolated from patients with SR and AF reported that compared patient with SR, miR-223-3p expression levels were downregulated in circulation expsomes of patients with AF (47). Thus, when the sample size used is relatively small and the reported expression of the gene is inconsistent with the literature, the selection offset should be expanded. Subsequently, further verification using larger sample sizes and multi-center research investigations is required.

In summary, the present study used microarray data on AF to identify significantly differently expressed miRNAs, and the results were subsequently verified in RAA tissues from patients with AF. Amongst the 43 significantly differently expressed miRNAs identified, miR-223-3p was discovered to be significantly upregulated in AF. Through GO functional term and KEGG signaling pathway enrichment analyses of the target genes of miR-223-3p, the ‘AMPK signaling pathway’ was identified as an important pathway. In addition, lncRNAs, including KCNQ1OT1, GAS5 and MINCR, were identified as targeting miR-223-3p. Furthermore, the results from dual luciferase reporter assay confirmed the existence of a targeted relationship between KCNQ1OT1 and miR-223-3p. These findings suggested that KCNQ1OT1 and GAS5 may serve crucial roles as ceRNAs in AF. These findings provided a solid theoretical basis for future intervention experiments and offered some potential novel targets for AF intervention. However, further transfection experiments both *in vivo* and *in vitro* should be performed to verify the findings from this study.

## Figures and Tables

**Table I.** Sequences of the primers used for reverse transcription quantitative PCR.

Target	Sequences (5′-3′)
hsa-KCNQ1OT1	
Forward	GAACTCTGTTTTGTTTTCTGCTGC
Reverse	CATAAATCCTGAAACAGACCCACTT
hsa-GAS5	
Forward	AAGCCATTGGCACACAGGCATTAG
Reverse	AGAACCATTAAGCTGGTCCAGGCA
hsa-MINCR	
Forward	CAGAAGAGCTTCATCGGCCC
Reverse	TCACAGACGCACTCTTCCCA
U6	
Forward	CTCGCTTCGGCAGCACA
Reverse	AACGCTTCACGAATTTGCGT
GAPDH	
Forward	GGAAGCTTGTCATCAATGGAAATC
Reverse	TGATGACCCTTTTGGCTCCC
hsa-miR-196b	
Forward	CGGGCTAGGTAGTTTCCTGT
Reverse	CAGCCACAAAAGAGCACAAT
hsa-miR-642b	
Forward	AGACACAUUUGGAGAGGGACCC
Reverse	GAGUGUGCCCAAGAGAAAGUUU
hsa-miR-3164	
Forward	TGTGACTTTAAGGGAAATGGCG
Reverse	CTCTACAGCTATATTGCCAGCCAC
hsa-miR-223-3p	
Forward	TGTCAGTTTGTCAAATACC
Reverse	AACTGGTGTCGTGAG
hsa-miR-425	
Forward	ATGACACGATCACTCCCGTTG
Reverse	GTGCAGGGTCCGAGGTATTC
hsa-miR-122	
Forward	GCGTGATGGAGTGTGACAAT
Reverse	GTGCAGGGTCCGAGGTATTC
hsa-miR-483-3p	
Forward	GGTGTCACTCCTCTCCTCC
Reverse	CAGTGCGTGTCGTGGA
hsa-miR-33b	
Forward	GTGCATTGCTGTTGCATTGC
Reverse	GTGCAGGGTCCGAGGT

miR, microRNA; GAS5, lncRNA growth arrest specific transcript 5; KCNQ1OT1, lncRNA KCNQ1 opposite strand/antisense transcript 1; MINCR, lncRNA MYC-induced long non-coding RNA.

**Table II.** miRNAs with increased expression in patients with atrial fibrillation.

Target	|Log2FC|	t	P-value
hsa-miR-33b	0.869586221	2.365376187	0.02954409
hsa-miR-483-3p	0.817867704	2.597063925	0.018293799
hsa-miR-122	0.772442509	2.541789208	0.020534659
hsa-miR-425	0.75856261	3.360950977	0.00351163
hsa-miR-196a	0.708341176	2.607334223	0.017903851
hsa-miR-2276	0.660417901	2.909299897	0.009413676
hsa-miR-493	0.649498855	3.344417162	0.00364197
hsa-miR-4271	0.63060241	3.202575415	0.004974358
hsa-miR-1539	0.622510073	2.536416632	0.020765806
hsa-miR-4296	0.607087624	3.262255063	0.004363723
hsa-miR-587	0.606329463	4.071871233	0.000726406
hsa-miR-4298	0.602845228	2.412892937	0.026808013
hsa-miR-548p	0.574869493	4.30723264	0.000431555
hsa-miR-605	0.550447818	2.738410535	0.013572845
hsa-let-7i	0.549892958	3.102281544	0.006194148
hsa-miR-621	0.541751345	2.533866076	0.0208764
hsa-miR-3125	0.538749883	3.107985712	0.006117547
hsa-miR-3915	0.534540407	2.802455828	0.011840557
hsa-miR-3180	0.528461253	2.632995349	0.016963765
hsa-miR-492	0.52778329	3.813405954	0.001289044
hsa-miR-193b	0.506441031	2.255811103	0.036874622
hsa-miR-641	0.505252296	2.308449626	0.033164494
hsa-miR-127-5p	0.500711212	4.461170028	0.000307439

miR, microRNA; FC, fold change.

**Table III.** miRNAs with decreased expression in patients with atrial fibrillation.

Target	|Log2FC|	t	P-value
hsa-miR-196b	−0.953061347	−2.387936306	0.028214045
hsa-miR-642b	−0.684669912	−5.232846332	5.78E-05
hsa-miR-3164	−0.667260096	−3.14912112	0.005591877
hsa-miR-223-3p	−0.667052957	−3.778345029	0.001393388
hsa-let-7e	−0.652894577	−3.358265208	0.003532485
hsa-miR-4264	−0.643875178	−3.129959531	0.005831009
hsa-miR-301b	−0.613918625	−3.965039772	0.000920629
hsa-miR-4254	−0.601866722	−3.137886674	0.005730892
hsa-miR-548o	−0.591841785	−4.056385355	0.000751777
hsa-miR-556-3p	−0.585672054	−2.622710761	0.017334758
hsa-miR-518c	−0.577279497	−4.166962591	0.000588435
hsa-miR-628-5p	−0.575507912	−5.543327212	3.00E-05
hsa-miR-518b	−0.57442567	−2.137014059	0.046696381
hsa-miR-130a	−0.570996268	−2.417688503	0.026545462
hsa-miR-3678-3p	−0.566643877	−2.618079507	0.017504327
hsa-miR-578	−0.556014824	−2.409518058	0.026994239
hsa-miR-889	−0.544245666	−6.228489463	7.35E-06
hsa-miR-433	−0.524268417	−2.193339731	0.041773836
hsa-miR-19a	−0.52202772	−2.739368993	0.013545215
hsa-miR-553	−0.520224796	−5.015733997	9.19E-05

miR, microRNA; FC, fold change.

**Table IV.** Clinicopathological characteristics of patients with atrial fibrillation.

Characteristics	SR (n=8)	AF (n=8)	P-value
Sex ratio, male/female	5/3	7/1	0.119
Age, years	53.125±8.839	54.125±7.338	0.907
Medical history			
Coronary heart disease (n)	3	1	0.569
Diabetes mellitus (n)	0	3	0.200
Hypertension (n)	5	4	0.334
Therapy			
CABG	3	1	0.569
AVR	0	2	0.467
MVR	5	3	0.619
Combined valve replacement	0	2	0.467

SR, sinus rhythm as control; AF, atrial fibrillation; CABG, coronary artery bypass grafting; AVR, aortic valve replacement; MVR, mitral valve replacement.

## Data Availability

The GSE68475 dataset is available from the Gene Expression Omnibus website (http://www.ncbi.nlm.nih.gov/geo/).
